# Greenspace Interventions, Stress and Cortisol: A Scoping Review

**DOI:** 10.3390/ijerph18062802

**Published:** 2021-03-10

**Authors:** Reo Jones, Robin Tarter, Amy Miner Ross

**Affiliations:** School of Nursing, Portland Health & Science University, Portland, OR 97239, USA; tarter@ohsu.edu (R.T.); rossam@oshu.edu (A.M.R.)

**Keywords:** biomarker, cortisol, greenspace, greenspace intervention, forest-bathing, horticulture therapy, nature-based intervention

## Abstract

Background: Engaging with nature can profoundly impact psychological and physiological health of persons across the lifespan. Greenspace interventions (GSI) encompass a broad range of strategic, nature-based activities for overall health and wellbeing. Within the past 20 years there has been a growing interest in the access to and management of greenspace to mediate the deleterious impact of acute and chronic stress, particularly, physiologic biomarkers of stress such as cortisol. Objective: This review aims to describe the impact of greenspace interventions on cortisol, to present the current state of the science on GSIs as they impact cortisol, and to uncover any limitations of current research strategies to best inform future research. Methods: A scoping methodology was conducted to systematically study this emerging field and inform future research by mapping the literature based on the GSI category, interventional design, cortisol metrics, and subsequent analysis of cortisol. Conclusion: Considerable heterogeneity in research design, aim(s), interventional strategy, and cortisol metrics were identified from a total of 18 studies on GSIs and cortisol outcomes. While studies demonstrated a potential for the positive association between GSIs and stress relief, more rigorous research is needed to represent GSIs as an intervention to mitigate risks of stress.

## 1. Introduction

The health benefits of greenspace have garnered much attention in the past decade with growing enthusiasm for the stress reducing properties of greenspace interventions. A 2018 meta analyses of 143 observational and interventional studies of greenspace exposure by Twohig-Bennett and Jones [1] discovered that varying degrees of greenspace exposure were significantly associated with decreased salivary cortisol levels (−0.05 (95% CI −0.07, −0.04)). A 2017 population-representative U.S. study demonstrated that the majority of adults and children viewed exposure to greenspace as beneficial to their health and wellbeing, yet perceived significant barriers to accessing greenspace, or greenspace related programming [2]. In addition to evidence from public opinion, the physiological and psychological benefits of spending time in nature, or greenspace, have received growing scholarly interest in the past decade [1,3,4,5,6,7]. Much of the extant research on greenspace interventions (GSI) focuses on the association between nature and mental health and wellbeing across the lifespan [3,7,8,9,10,11]. Several reviews focusing on the physiologic benefits of greenspace have included outcomes related to stress including allostatic load [12], cardiovascular disease biomarkers [13] immune function [14] and cortisol [15]. Few reviews have examined both the methodology of GSIs, and the processes by which specific physiologic outcomes are measured.

One critique of greenspace literature is the extraordinary heterogeneity within the interventional terminology, use of methodologies of measuring impact, and inconsistency in observed health-outcomes [16,17]. Forest bathing, gardening, and park visits are often described as either “nature-based” or “greenspace” interventions, despite the unique operationalization of these practices within the literature [18]. For the purposes of this review, greenspace interventions (GSI) will be considered a subset of nature-based interventions (NBI), which is consistent with well-cited current literature [1,10,16,19,20]. A NBI is described as an activity or process aimed to engage people in natural settings such as parks, forests, mountains, beaches, gardens, and savannas, with the goal of improving health-related outcomes for persons across the lifespan [18,21,22,23]. NBIs (including GSIs) are usually intended to increase the level of exposure to, or contact with, the natural environment [21,22,24]. GSIs generate a multitude of health benefits along multiple pathways [24] through a variety of potential mechanisms [25]. While the term greenspace is applied inconsistently across various disciplines, in the context of this review greenspace is most defined as inclusive of outdoor environs with some form of vegetation such as forests, gardens, prairies, woodlands, with “green” foliage as distinct from savannas, i.e., brown-spaces, or bodies of water, i.e., blue-spaces [26]. GSIs are NBIs, and therefore they are nested within the construct of NBIs [27]. In the context of urbanicity, the World Health Organization defines GSIs as codified programs which can be carried out in large, vegetation-laden, accessible outdoor, natural spaces [28]. GSIs are considered dynamic, community-based, and involve human exposure to uncontrolled, natural, green, outdoor environs through unique procedures and processes [4,10,29].

Existing studies have investigated the myriad health benefits of GSIs. However, research on specific physiologic benefits of GSIs through the stress recovery pathway is limited. The impact of GSIs on cortisol was chosen as the focus of this review because cortisol is a widely studied biomarker frequently used as a measure of human stress levels [30,31]. As aforementioned, significant positive associations between GSIs and decreased peak cortisol levels have been demonstrated by several studies in a variety of natural settings [1,5,15,30,32]. Akin to the great diversity of approaches to GSIs, much variation exists in the ways in which cortisol is measured and analyzed. However, it should be noted that based upon study design and intended outcome, different cortisol measurement approaches are valid and reliable proxies for stress [30]. Therefore, the aims of this review are, 1. To describe the ways in which the impact of GSIs on cortisol have been studied, and 2. To present the current state of the science on the impact of GSIs on cortisol, and 3. To uncover limitations of current research strategies in an effort to inform future research on GSIs and stress-sensitive biomarkers.

## 2. Materials and Methods

### 2.1. Identifying the Research Question

To address these aims, Arksey and O’Malley’s scoping review methodology was chosen to systematically identify, explore, and study the range of extant literature on GSIs, stress and cortisol in an effort to delineate our key research question [33]. Given the variety of interventions and outcome domains pertaining to this topic, a scoping review is the most appropriate methodology to explore this growing field. Our scoping process followed the five steps, or stages outlined by Arksey and O’Malley within their adapted scoping review framework [33]. These stages include (1) identifying the key research question, (2) searching for relevant literature, (3), selecting studies based on the necessary criteria, (4) charting, or graphically representing crucial detail from the selected studies, and (5) aggregating, summarizing, and reporting the findings [33]. In addition to following these five stages, we based the aims of this paper on a proposed research priority for studying the health effects, or benefits of nature as outlined by Frumkin, Bratman [25]. In their Delphi expert elicitation study, authors Shanahan, Astell-Burt [22] recommended that in order to integrate NBIs and GSIs into the mainstream, we need to better shape our research priorities by discovering key gaps to implementation and translation. Based on systematic review of the state of the literature, Frumkin, Bratman [25] proposed seven domains for studying nature contact and health in an effort to address these critical next steps in advancing the field. Of the seven, our review focused on domain (1) *Mechanistic and Biomedical Studies*, by identifying mechanistic biomedical research homing in on physiological pathways relating to the observed effects of nature-based interventions.

This review did not incorporate any systematic appraisal tools for critically evaluating the quality of the literature. Such quality appraisal is not a part of the scoping directive within the chosen scoping methodology [33]. Rather, the literature was explored with the intent of demonstrating what is presently known and what limitations need to be addressed further to inform future directions for rigorous scientific research on GSIs and cortisol.

### 2.2. Identifying Relevant Studies

Driven by the nature contact research domain, Mechanistic and Biomedical Studies [25], as well as the recommendations from the work of Shanahan, Astell-Burt [22], this scoping review was directed by the nature of its’ aims to explore both how research is performed and what has been discovered about the impact of GSIs on cortisol. Inclusion criteria were: empirical articles, author ascribed experimental designs, designs which included a manipulation, quasi-experimental studies of GSIs measuring participants’ cortisol levels as potential outcomes of the intervention [34]. Only articles available in English and published in peer-reviewed journals between 2010–2020 were included to retain the most prescient literature. Exclusion criteria included: Observational studies, epidemiological research, commentaries, reviews, protocol papers, and grey literature were excluded on the basis of the study’s aim of examining interventional research solely with reported results. While review articles were excluded from final selection, the reference lists of these articles were manually searched to identify literature meeting our eligibility criteria [33].

Authors of current systematic reviews on NBIs to date have noted that determining appropriate search terms for GSIs can prove challenging given the heterogeneity of the terminology for these interventions [35]. GSIs have been studied widely across the globe and in a variety of settings, therefore attention to semantic detail is critical, however, overly specific search terminology may lead to unintended omissions given the rapid growth and change in this field of research [10,16,26]. Searching for peer-reviewed texts in academic journals involved multiple rounds of strategizing with keywords and subject terms. Keyword searches were performed in all relevant databases that had controlled vocabularies. The available databases often had no equivalent subject terms for relevant keywords. Therefore, keyword equivalents were used in the final search, and lists of keywords were drawn from two recently published large systematic reviews of GSIs. [10,15]. The search terminology utilized in this review is represented in Table 1.

### 2.3. Study Selection

**Figure 1:**
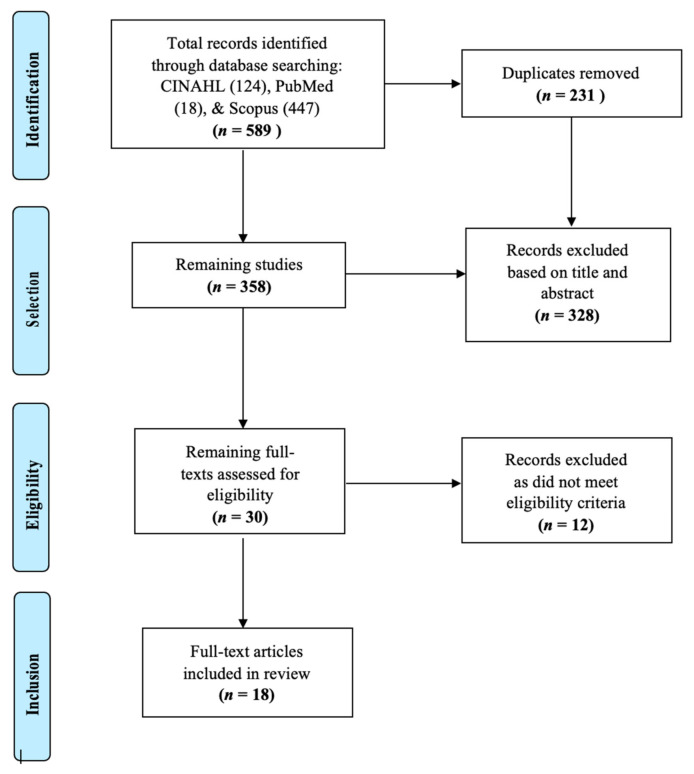
Flow diagram of search history and resulting articles.

According to Arksey and O’Malley, [33] the initial scoping process for study selection should be as comprehensive and broad as possible. Therefore, we chose three main, highly indexed databases for our initial search. Searches of the Cumulative Index of Nursing and Allied Health Literature Plus with Full-Text (CINAHL), Scopus, and PubMed (MEDLINE) resulted in the identification of 589 records which were collected into a citation manager. Duplicates were removed, resulting in a total of 358 articles retained for screening. A total of 328 articles were removed based on the inclusion and exclusion criteria from examination of titles and abstracts for key words, and 12 additional articles were removed for not being full-text articles, resulting in the final inclusion of 18 articles as represented in Figure 1. While searched for, no novel literature pertaining to the scope of this review was identified through manual reference list searches.

## 3. Results

### 3.1. Charting the Data

Per stage 4 of the Arksey and O’Malley framework, the included studies (*n* = 18) were charted and sorted into a literature matrix based on the study location, population, research design, type of intervention, type and timing of cortisol measure, and study findings (Table 2) [33]. The synthesis of included literature was organized into relevant categories based on the type of GSI following the typologies of NBIs specified and operationalized in the 2019 Delphi Study [22] and further supported in a seminal 2020 review of GSIs [10]. Given that cortisol was our primary outcome of interest, we included its’ relevant metrics alongside resulting significance within our table and in the discussion following. The following table represents a comprehensive outline of the synthesized literature on GSIs and cortisol outcomes, grouped by interventional design specific to Horticulture Therapy (HT), Forest Bathing (FB), Greenspace Exposure (GE), and Outdoor Activity Programming (OAP).

All studies included in the synthesis section of this review aimed to determine the impact of GSIs on cortisol. While cortisol can be measured to understand a variety of disease pathologies [30], in all included articles, it was studied solely as an indicator of physiologic stress, and under the presumption that GSIs have a causal association with variability in cortisol levels. Results of this study demonstrated that NBIs defined by the Delphi Study [22] as “treatment” or “prevention” interventions could be grouped together with NBIs that intend to “change behavior” or moderate psychological and physiological outcomes. Each of the included GSIs fit within the typology determined by the aforementioned Delphi panel’s 5 categories of: horticulture therapy (HT) and gardening; forest bathing (also known as Shinrin-yoku in Japan); greenspace exposure (GE); outdoor activity programming (OAP) such as hiking or wilderness games, “green” exercise, and forest schools; and park prescriptions (PP) [22]. In addition to outlining the typologies of interventions and their impact, we provide a discussion of cortisol and its’ meaningfulness within our selected studies. Given the myriad ways in which cortisol was integrated into the literature, as well as its’ dynamic complexity as a biomarker of stress, we provide a synthesis paragraph describe it below as our main outcome of interest.

### 3.2. Collating, Summarizing, and Reporting the Results

Based on the Arksey and O’Malley framework, the scoping process is geared towards presenting an overview of selected studies within, does not seek to synthesize evidence to the depth of a systematic review, and is geared towards representing the weight of the evidence as it relates to the research question [33]. Further, it is recommended that reviewers expound upon the charted data (see Table 1) by providing greater explication and attention to detail relevant to the studies and their findings. Therefore, we have organized our Results sections according to our primary outcome, cortisol as a biomarker of stress, as the dominant variable within the selected studies’ aims, as well as each interventional typology, presented in our table.

#### 3.2.1. Cortisol Metrics

Cortisol, a glucocorticoid (steroid) hormone released by the adrenal cortex via the hypothalamus-pituitary-adrenal (HPA) axis, is considered a valid and reliable biomarker of physiologic stress and can be easily measured from saliva, urine, hair, blood plasma, and serum [54,55]. The way in which cortisol is measured, depending upon the tool and process of extraction, can greatly impact the reliability of results, [56,57,58,59]. El-Farhan, Rees [57], noted that despite serum cortisol being the “analyte of choice,” in stress research, many immunoassays have been compromised by poor standardization and specificity. Segerstrom and Miller [60], suggested that intra-individual variability in cortisol levels may require more than 10 days of consecutive data collection to yield reliable measures. Cortisol measurements from bodily fluids are not always reflective of long-term stress exposures, whereas hair cortisol concentrations (HCC) may be preferred to measure serial cumulative cortisol exposure, to set a baseline for individual sample variability, or for longitudinal study designs [61]. Poll, Kreitschmann-Andermahr [62] compared *Salivette* cortisol measures with total and free serum cortisol to determine the most reliable metric and found that *Salivettes* (cotton swabs with which saliva is absorbed), are the most consistently accurate. A recent study comparing the validity and reliability of the gold standard, validated, Enzyme Linked Immunosorbent Assay (ELISA) salivary cortisol collection kit with a more novel real-time, participant-collected field-based kit, showed that newer methods for immediate cortisol assessment are critical given the time-sensitive nature of sample collection. In addition to the variation in collection processes and analyses, individual variability and complex environmental factors play a significant role in cortisol production, problematizing the accurate measurement of cortisol [54].

Of the 18 studies included in this review, 13 measured salivary cortisol, one measured both salivary and serum cortisol, and two measured plasma cortisol. No studies within this review measures or mentioned HCC. A systematic review and meta-analysis [15] on the effect of forest bathing on cortisol levels, noted that while cortisol is a reliable biomarker for stress, in order to properly study cortisol for medical purposes, a patient’s age, gender, daily routine, lifestyle characteristics and behaviors should be taken into consideration alongside the stressor or intervention of a study (p. 1118). Diurnal cortisol rhythms are typically higher upon waking, increase further about 30 min after waking, then sharply drop after the awakening hour and slowly decrease until nighttime [30]. Therefore, time of day as well as genetic factors, psychosocial determinants of health, mental and physical health status, and personal daily behavior have extraordinary impacts on individual cortisol differences [55]. Studies in this review represent a broad sampling of cortisol metrics, with only one study [50] measuring the diurnal cortisol response trajectory in relationship to GSIs.

#### 3.2.2. Horticulture Therapy

A total of four studies utilized horticulture therapy (HT) as their intervention strategy [36,37,38,39]. None of the studies in this category employed a theory or framework to ground their methods, but each study defined HT similarly. HT as described by Detweiler, Self [36], “applies the art and science of growing plants to improve physical, mental, and spiritual well-being,” and can be utilized to “contribute to restoration from stress,” (p. 37). Researchers Ng, Sia [38] defined their HT protocol as a “gardening” intervention designed by a HT trained instructor with outdoor sessions in parks, gardens, nature reserves, and the incorporation therapeutic guided walks. Another field experiment suggested that their “stress-relieving” and “restorative” intervention was intended for participants to engage in “light” gardening activities [39]. Han, Park [37], utilized a structured HT program in their controlled experiment, but did not include “walking” as part of the intervention. Much like the waitlist controlled trial by Ng, Sia [38], authors Han, Park [37], incorporated a series of systematically organized activities carried out by horticulture therapists. There was a considerable range of activity levels in each of the HT interventions reported in the literature; however, physical activity (PA) was not controlled, or reported as a potential confounding variable regarding cortisol for any of these studies.

In their pilot study, Detweiler, Self [36], compared the impact of HT against Occupational Therapy (OT), on stress-related outcomes among veterans in a standardized 28-day treatment program. Participants were instructed not to “participate in any activities that might incite strong emotions,” and were prohibited from eating, drinking, or smoking an hour before data collection so as not to interfere with cortisol production (p. 38). Researchers Van Den Berg and Custers [39], also noted their participants were to abstain from eating, drinking, or smoking up to 2 h before data collection. Neither the Detweiler, Self [36], nor theVan Den Berg and Custers [39] offered justifications as to why these parameters were included for their participants. Researchers Ng, Sia [38] and Han, Park [37] did not list or cite specific parameters for participant behavior pre-cortisol sampling.

All studies in the HT category collected cortisol pre and post intervention. However, cortisol sampling time-frames varied significantly within and between studies. In Ng, Sia [38], plasma derived cortisol was collected for both the initial HT group (active intervention) and the waitlist control in the morning at baseline, 3 months and 6 months. Researchers used an rANOVA to examine the difference between active intervention and control groups, with three time-points entered as within-participant factor (*Time*), resulting in a significant interaction between time (between groups difference), and a significant main effect of time (within groups difference). However, there were no significant effects of *Time*, or *Group* or *Time with group* interactions on plasma cortisol values (*p* < 0.05) [38]. Detweiler, Self [36] tested salivary cortisol at baseline and the culmination of weeks 1, 2, and 3 of their intervention, but had missing data from a lack of sufficient self-collected saliva for the control with non-significant results (*p* = 0.43, n.s.). By contrast, Han, Park [37] and van den Berg, Wendel-Vos [63] demonstrated significant results when comparing different time points during the intervention. Via paired-t-tests, Han, Park [37], reported that mean cortisol levels decreased significantly in the HT group, from pre- to posttest (*p* < 0.05), while no significant differences in cortisol levels were observed in the control group from pre- to posttest.

In Van Den Berg and Custers [39] cortisol decreased significantly in the gardening group from post-stressor to post-activity, (*p* < 0.001) as well as the reading group, (*p* < 0.05), however, the specifics of the stressor, described as the stressful task, apart from the experimental intervention, were not described (p. 6). Unique to this category, Van Den Berg and Custers [39] studied the association between cortisol level change and positive mood states (POMS). The results of which were significantly, negatively correlated in the gardening group (*p* < 0.05), significantly positively correlated in the reading group (*p* < 0.05), and with the difference between both correlations being significant (*p* < 0.01) [39]. All studies within this category noted limitations to their results with regard to sample size, confounding variables of time, setting, extraneous and unaccounted for activity, as well as the lack of true controls [34].

#### 3.2.3. Forest Bathing

The impact of forest bathing (FB) on physiologic biomarkers of stress have been widely studied in research utilizing observational, descriptive designs. The four articles included in this review represent a small, homogenous sample of the literature in the context of GSIs with forest bathing [41,42], or forest therapy-based interventions [40,43].

Kobayashi, Song [40] described FB as an internationally used term “to refer to forest exposure for therapeutic or preventive health purposes,” (p. 1). Of these studies, three ascribed a theoretical lens denoting how, or why forest-based interventions are healthful [40,41,43]. Lee, Park [41], referenced E. O. Wilson’s *Biophilia Hypothesis* [64], Kaplan’s Attention Restoration Theory (ART) [65], and the psycho-evolutionary theory [66], all of which support the idea that positive associations with nature may stem from environmental familiarity and its’ potential to have a “restorative” impact on physiologic and psychologic functioning. Citing psycho-evolutionary theories on the restorative and effects of nature, Sung, Woo [43], suggested that forested-environments increase “directed-attention abilities,” and that forest-laden regions provide a “psychologically familiar,” or “comfortable milieu,” based upon the idea that humans evolved alongside green-forested surroundings. Kobayashi, Song [40] referenced the *Biophilia Hypothesis* [64], but from the dual perspective that natural environs can be calming and/or stressful depending upon an individual’s developmental background- the natural settings comprising their life-history. Mao, Lan [42] referenced the importance of evolutionary history pertaining to the psychological and physiological benefit humans feel in forested regions yet did not cite a theory. All of the studies in the FB group were performed in the forested regions of Southeastern Asia, yet only one study [40] noted that the assumption driving the health benefits of forested environs for all falls short when considering global populations and different landscapes.

All four field studies measured cortisol level change in two main participant conditions—A *forest group* where forest bathing took place in a designated forested area, and an *urban group* (functioning as the control) where participants were situated in a city-center. Referencing the higher incidence of stress related disease and general poor health associated with rapid urbanization, Lee, Park [41] and Mao, Lan [42] maintained that the immune-modulating aspects of forest bathing could be invaluable as a preventative medicine intervention. To this effect, Mao, Lan [42], Lee, Park [41], and Sung, Woo [43], gave extensive explanations of their study sites including landscape, topography, regional access, as well as the particular species of trees in the forested regions.

Noting that physical activity can have significant impacts on cortisol levels, Mao, Lan [42], sought to “investigate and distinguish between the effect of walking and that of forest exposure on salivary cortisol concentration,” in urban and forested environs (p. 4). To this effect, they standardized several features of their intervention to ensure accurate cortisol readings such as: having participants rest pre-baseline cortisol measures and walk at a slower place on smooth terrain during the walking phase of the intervention. Lee, Park [41], instructed participants to remain seated for 15 min after arriving at each study site before baseline cortisol measures, which “mitigated the physiological effects of physical activity before measurements,” (p. 94). Lee, Park [41] and Kobayashi, Song [40] noted the regulation of food, alcohol, and stimulant intake pre- cortisol sampling for their participants, while other studies in this category did not. Sung, Woo [43] implemented a multifaceted, prescribed forest therapy program, which incorporated a series of cognitive and behavioral techniques, yet did not control for nutritional intake or PA.

Studying the impact of walking on salivary cortisol levels in urban and rural settings, Kobayashi, Song [40] found a significant interaction effect between environmental setting and walking (*p* < 0.001), with mean salivary cortisol significantly lower in the FB group than the urban walking group (*p* < 0.001) from pretest to posttest. Researchers noted that time passing as well as intense walking can confound cortisol results, suggesting that their study (25 min in each setting over 2 days), is more appropriate for the field (p. 4). By contrast, Lee, Park [41] participants were passive, or seated in the exposure conditions, which resulted in a positive effect of forest viewing stimuli on study participants. Salivary cortisol levels were significantly lower in the FB group compared to the urban viewing group (*p* < 0.05) at baseline and just before the stimuli (*p* < 0.01). Results post-stimuli were non-significantly different between conditions, but researchers noted that lower salivary cortisol levels in the FB group compared to the control group may be due to “participants feeling relaxed or less stressed in the forest environment” as well as, “an innate desire to interact with natural environments,” (p. 98).

Comparing pretest and posttest data, Mao, Lan [42], determined that serum cortisol values were significantly lower in the FB group than city-site (*p* < 0.05), which they attributed to the stress-reducing impact of greenspace compared with city environments. As with Sung, Woo [43], salivary cortisol level reduction was significantly larger in the forest group than the control group (*p* < 0.05) at the culmination of their 3-day intervention, with posttest measures taken week 8. As with the HT group, studies in this category had limitations including small sample sizes, unmeasured variation in lifestyle factors, preexisting preference for natural settings among participants, and lack of true controls.

#### 3.2.4. Greenspace Exposure (GE)

Some of these studies in this category incorporated *active* (such as walking), or *passive*, (sitting) interventions, see Table 2. These distinctions were evident in the study aims to determine if nature exposure had a significant impact on physiologic stress [44] or if controlled physical activity (in green or urban settings) could decrease cortisol levels [45]. Researchers Olafsdottir, Cloke [47], aimed to disentangle the effects of nature exposure and exercise in their comparison of nature walks, videos of nature, and treadmill walking. Another two studies aimed to determine how the connection between urban greenspace and positive affect impacted cortisol [46,49]. Razani, Morshed [48] addressed how *active* nature exposures, such as park prescriptions, could decrease physiologic stress experienced by low-income families with limited access to the outdoors.

No studies in this category grounded their research methodology in theories or frameworks. Yet, three articles cited theory in their literature review as preexisting evidence to support their study protocol and outcome selection. Beil and Hanes [44] and Olafsdottir, Cloke [47] reference the aforementioned PES theory [66,67] and ART coined by Kaplan and Kaplan [65] with regard to how nature promotes health and wellbeing, and Tyrväinen, Ojala [49] referenced ART in relation to their choice for utilizing specific psychological scales in their study design. Beil and Hanes [44] justified the non-significant negative trend of their cortisol results as congruent with the PES model- asserting the *Very Natural* environment was more restorative.

The parameters and justifications for cortisol collection processes varied significantly between studies. Studying passive nature exposure, researchers Beil and Hanes [44] noted how and why they intentionally controlled participants’ physical activity before collecting salivary cortisol swabs. Studying active nature exposure, Grazuleviciene, Vencloviene [45] instructed participants not to consume stimulants or any food before their data collection, but did not cite the rationale. Olafsdottir, Cloke [47], noted that *not* controlling dietary intake, or measuring diurnal cortisol rhythms were limitations to their study, that offset the fact that their interventions were scheduled in the afternoon to standardize the circadian rhythm of cortisol. Studies by Mokhtar, Abdul Aziz [46], Razani, Morshed [48], and Tyrväinen, Ojala [49] did not include specific behavioral parameters related to cortisol collection, but noted, in accordance with the other studies in this category, that collection was time-sensitive. Cortisol collection methods were entirely heterogenous within the GE group, but this is unsurprising given the variety of interventional designs and the type of nature exposure within each study.

Grazuleviciene, Vencloviene [45], investigating the restorative effects of walking in nature compared to urban streets in post Myocardial Infarction (MI) patients, noted that greater negative affect was associated with higher mean cortisol levels (*p* < 0.05), and that cortisol slope was negatively associated with heart rate (HR) increases and blood pressure changes from day 1 to day 7 of their study (*p* < 0.05). This finding suggests that negative affect is a greater predictor of stress in post MI patients than positive affect (which was non-significant). Mokhtar, Abdul Aziz [46] found that their urban greenspace intervention significantly lowered mean cortisol between pre- and posttest (*p* < 0.05), while levels in the participants who walking in the city center experienced in increase in mean cortisol (*p* < 0.05). Olafsdottir, Cloke [47] and Razani, Morshed [48], also noted significant decreases in mean cortisol levels in their participants from baseline to posttest. Tyrväinen, Ojala [49], noted a significant main effect of time on cortisol between pre- and post- exposure, but found a non-significant interaction between study setting and time, and suggested that cortisol decreased over time (from baseline, through the intervention and posttest and again at3hrs. post intervention), independent of study setting. No other study in this section tested the interaction of time of sample collection and cortisol levels.

#### 3.2.5. Outdoor Activity Programs

Only one of the four studies incorporating exercise in the outdoors referenced theory as foundational to their study design. Chang, Davidson [51] described how PET [66] and ART [68] guided their hypothesis that *green exercise* can decrease stress response by improving college students’ ability to maintain focus through cognitive capacity building with positive associations from nature-based activities. Chang, Davidson [51] randomized three groups of college students who volunteered for a three-day field trip involving canoeing, kayaking, and backpacking, as a potential antidote for stressful scholastic activities. Calogiuri, Evensen [50], also studying the impact of green exposure paired with exercise on stress, or the “synergistic benefit in adopting physical activities while at the same time being directly exposed to nature,” (p. 100), studied job-related stress in a workplace setting with the intention of incorporating an outdoor exercise program that was accessible to employees. Both Chang, Davidson [51] and Calogiuri, Evensen [50], designed their interventions with the intention of creating a sustainable, reproducible practice for populations vulnerable to the complications of long-term stress exposure (college students and office-based workers, respectively).

In their prospective longitudinal survey, Dettweiler, Becker [52], aimed to study the impact of a nature-based outdoor scholastic program on 5th grade students during peak months of academic stress. Their aim was to see if adding curriculum based in an outdoor forested region was more impactful on children’s diurnal cortisol rhythms than traditional classroom settings while controlling for physical activity. While, Niedermeier, Grafetstätter [53] did not aim to study a stress-burdened population, their intervention protocol was relatively similar to studies in this category comparing active outdoor programs with passive controls [50,51,52]. For example, Niedermeier, Grafetstätter [53] studied the acute and long-term impact of mountain hiking, or green exercise, indoor exercise, and sedentary behaviors (control), on physiologic stress to determine if indoor vs. outdoor activity impacted salivary cortisol differently than indoor sedentary behavior. For each outcome measure, three x two repeated measures ANOVAs were used to analyze the effects of each study condition (i.e., green exercise, or indoor exercise), on time, and time-by condition interactions. Results demonstrated a significant effect of time, with cortisol concentrations decreasing over time in all study conditions, as well as a significant interaction between time and study condition [49]. Via linear mixed effect model of log cortisol data, Dettweiler, Becker [52] also found a significant effect of time on cortisol, and that children who spent more time in outdoor scholastic programming had a significantly greater decline of cortisol concentration when compared to the control.

Of the four outdoor activity program studies, only one study addressed the potential for confounding variability in cortisol results in relationship to nutritional intake and physical activity [50], which may have been due in part to the duration of the studies and challenge of controlling for extraneous variables by the nature of the studies’ designs. For example, Calogiuri, Evensen [50] imposed a 45 min exercise routine indoors and outdoors, Dettweiler, Becker [52] incorporated an outdoor educational programs and activities which not only gave students the opportunity to elect play and activity based on their desires, but also engaged students in planned activities in the forest (p. 4). While physical activity was largely unstructured in the Dettweiler, Becker [52] study, it was captured by accelerometer measurement and studied as an interaction term along with cortisol. Chang, Davidson [51] suggested that lacking a true control group was one potential limitation their study along with the inability to control for variance in scholastic activities of the students (as the intervention occurred during the school year). Additionally, data collection took place in the evening, which did not account for their participants’ individual diurnal cortisol cycles. The nature of each interventional activity was considerably different between studies (p. 79).

The study by Niedermeier, Grafetstätter [53] is unique in that they analyzed salivary samples through the passive drooling method pre- and post-exposure and described the process of sample absorbency with detailed accuracy regarding individual’s sampling variance, reported as intra- and inter-assay coefficients of variation, (p. 4). Their study resulted in a significant decrease in mean salivary cortisol across all conditions over time (*p* < 0.001), while cortisol decreased to a greater extent after hiking and treadmill walking than in the sedentary control, with no significant difference between mountain hiking and treadmill walking [53]. Chang, Davidson [51], also studying time as an interaction term, found no significant main effect between sex and time, or activity and time on cortisol, but noted that cortisol levels decreased significantly at the beginning of the interventions with a mild trend upwards during the intervention and posttest.

Additionally, unique to this review, Calogiuri, Evensen [50], sampled, or labeled, salivary Cortisol Awakening Response (CAR), as their baseline to compare against pretest and posttest measures of serum cortisol. Results from Calogiuri, Evensen [50] suggest that while there were no significant differences between groups for CAR and serum cortisol concentrations over 3 time points, inter-individual differences in CAR between the participants were very large. CAR measurements showed quite large inter-individual (SD 124.97, 108.42 and 110.15), and mean CAR levels that decreased significantly from pre-to post exposure for the outdoor group compared to the indoor control, (p.106). Dettweiler, Becker [52] reported that their intervention group (named the *Outdoor Learning Program*)*,* also had a greater overall decline in cortisol when compared to the control posttest (*p* < 0.009), with lower levels reported in the spring season- citing weather as a potential confounder. They intended to measure mid-program and long-term buffer effects of cortisol in their population with HCC, but these data could not be analyzed due to a lack of sufficient sample from incomplete participant sampling, and researchers suggested diurnal cortisol rhythms need to be studied in future research. As with the other GSI classifications, studies in this category noted limitations with regard to the challenges of small sample size in purposive sampling as well as the inability to control for significant extraneous variability in activity levels, or issues inherent to study setting such as weather-related change.

## 4. Discussion

Included in this review are a total of 18 articles representing four categories of GSIs and various cortisol outcomes. This body of literature represents a diversity of approaches to cortisol collection and interventional strategy. Studies varied widely in terms of employing theoretical frameworks, sampling strategy and participant selection, cortisol measurement protocol and justification, as well as analysis and results. It should be noted that this review contained only studies employing an experimental design, in actual nature, with cortisol as a primary outcome specific to stress-response and recovery. There is a growing body of literature employing virtual greenspace methods for stress recovery among other positive health outcomes [69,70]. Correlational, observational, and epidemiological studies comprise the majority of the literature on GSIs and stress-related outcomes. While virtual interventions and epi-related studies did not meet the eligibility criteria for this review, they may offer critical insights into the mechanisms underlying GS, GSIs, and health. Elucidation of such mechanisms may support future endeavors to bolster GSI-methodology for future applications in clinical settings or the public health arena [25].

### 4.1. Heterogeneity of Study Design and Sampling Size

Of all 18 studies included in this review, only one by Chang, Davidson [51] included a theoretical framework as the basis for designing their intervention strategy. While a few other studies referenced prominent theories in the behavioral and psycho-evolutionary sciences as background to present-state literature, the overall lack of theory-driven research design is a significant limitation to the interpretation and function of the interventions and their resulting outcomes. Theory-driven research helps to formulate, explain, predict phenomena, and locates a research problem within the context of the field or phenomena. [34]. Given the relative novelty and exponential growth of the GSI field, theoretical frameworks could support future research and help readers to navigate causal claims to the specific efficacy of nature-based healing. Furthermore, theory-driven research grounds the reader with a philosophical lens with which to offer robust critique, thereby fortifying future methodologies. Given that theory was underrepresented in the selected studies, justification was lacking for specific methodological frameworks.

Most all studies had incredibly heterogeneous samples with only one reporting participant ethnicity Razani, Morshed [48], four reporting samples with either 100% male participants [40,41,42,46], or highly skewed gender in sampling, i.e., 23 males and 1 female [36], and few other studies with mostly female samples. The vast majority of sampling methods utilized were purposive or convenience, which does not allow for generalizable inferences. Additionally, the age-ranges of several study samples were fairly constrained. This was justified in the HT grouping, but not the FT group of literature. Life-course has a significant impact on stress-sensitive biomarkers and stress-recovery, but age as a confounder was not described in any of the studies presented here. However, some studies deliberately sampled for homogeneity such as the work of Detweiler, Self [36], sampled from a volunteer pool of PTSD diagnosed veterans preregistered in a treatment program, or Calogiuri, Evensen [50] sampled from a pre-determined workplace setting based on the nature and location of the work. Study populations in the Forest Bathing category were nearly 100% college age males, but authors did not present the rationale, or cite previous work for specifically choosing this population. While several studies did not give justifications for their population selection in their study design, few others specifically integrated populations in situations of high-stress burden. Studies were not selected for this review based on sample characteristics and the extent to which this review’s findings can be generalized to other populations. This is evident not only by sampling methodology, but the concurrent lack of attention to detail to inter- and intra-individual cortisol level differences based on natural diurnal cortisol level change.

### 4.2. Limitations of Cortisol Data Collection Methods and Analysis

As aforementioned, it is widely accepted that in order to understand an individual’s diurnal trajectory with regard to cortisol, several days (at least 10 or more) of repeated measures are necessary for consistency in analysis [62]. However, only one study specifically measured diurnal cortisol time points over several weeks [50], while one reported cortisol slope, but did not indicate which samples specifically were designated for this analysis [45]. Three studies in the FB group collected cortisol each day for three days, while one study in the GE group collected cortisol samples pre-post in a single day. These four studies either incorporated a waitlist control, or crossover design with 2 or more conditions, as seen in Table 2. However, no two studies truly compared in their cortisol collection methods and protocols, despite cortisol being the primary health outcome addressed. Such conflicting study designs, with multiple, repeated, yet different greenspace exposures may have had a significant impact on cortisol levels, but the extent to which the outcomes were a result of the specific interventions is unclear and therefore construct validity as well as generalizability is of great concern [34]. Future studies may wish to employ more robust serial cortisol measures, or include HCC, a more reliable approach to cortisol sampling for repeated exposures [61]. While cortisol is one biomarker for stress recovery, it is highly variable requiring several additional protocol steps to ensure covariates were accounted for. Future researchers are encouraged to study cortisol alongside additional stress-sensitive and pro-inflammatory biomarkers, as well as correlating biological measures with mental health outcomes [25].

Regarding cortisol assays, all studies mentioned the specific kit and functional tests used to measure their cortisol results. While the purpose of this review was not to study the validity and reliability of those specific test kits, all were known to the literature as widely accepted common tools of cortisol measures. However, not a single study referenced the reliability or validity of the tool used to collect cortisol, or the justification for the chosen methods. Furthermore, three studies noted participants collected their own samples, while 7 studies did not disclose who administered and collected cortisol sampling kits (saliva) or performed phlebotomy (plasms/serum). A total of 10 studies did not report standardization of time, specifically when cortisol levels were drawn, or sampled with respect to interventions lasting several days. A total of three studies reported *time* as an interaction term, despite *time* being represented in a number of studies as a critical deciding factor in cortisol sampling. For example, several studies mentioned baseline collections were performed in the morning hours just after peak diurnal cortisol level change. Given that *time* is a major factor in analyzing cortisol accurately [30], these omissions of *time* as a critical factor in analysis leading to concerns over statistical conclusion validity [34]. These limitations specific to cortisol data collection and analysis are not novel to the field. A 2019 systematic review and meta-analysis on the impact of forest bathing interventions on cortisol, noted significant heterogeneity specific to cortisol collection methods and analysis [15]. While GSIs may significantly reduce cortisol values, it is difficult to generalize these findings without more robust analysis. Furthermore, the majority of studies reported *p*-value level significance without effect sizes to inform readers about the degree to which these interventions were effective in reducing cortisol. A 2019 systematic review and meta-analysis investigating the effects of public GS on acute psycho-physiologic response echoed this concern regarding inconsistent cortisol measures and limited statistical reporting [71].

### 4.3. Limitations of This Review

Given concerns over study quality and the limitations of the scope of this review, the impact of GSIs on cortisol levels needs to be studied further. Specific attention should be given to unique greenspace intervention types with ongoing investigation of exposure properties. Future research endeavors should give specific attention to interventional terminology associated with NBIs and GSIs. Further, a systematic review of the literature is warranted. As contributions to GSIs and physiologic stress reduction in the literature continue to grow, future scoping reviews may endeavor to focus on one specific GSI with the biomarker cortisol to determine what, if any, crossover similarities are present.

Given that repeated physiologic stress exposure can be harmful to the overall health and wellness of persons across the lifespan [31], understanding how cortisol is impacted across a wide array of GSIs can contribute to the literature meaningfully. Further research must be conducted to best determine key features of GSIs and their impact on cortisol and associated stress-sensitive, pro-inflammatory biomarkers with confirmed reliability and validity of the tools used and of the tests analyzed. As a scoping review, this analysis endeavored to illuminate and describe the scope of the literature to date. Significant concerns over study quality and heterogeneity of methodologies presented as a key finding. A systematic review and meta-analyses would be an appropriate next step with the available and exponentially growing body of literature within the GSI and NBI field. Ongoing stringent quality appraisal of the literature will offer the field a more thorough investigation of known gaps and future directions for research.

## 5. Conclusions

This review was successful in determining how the impact of GSIs on cortisol have been studied while uncovering limitations of current research strategies. Further investigation must be done to present the current state of the research on the impact of GSIs on cortisol, and more broadly, the stress-recovery process via a variety of psychologic and physiologic pathways. The pandemic events of 2019 have presented humanity with an extraordinarily high stress burden including the physiologic and psychologic challenges of ongoing prescribed isolation. The 2019 emergence of the novel Coronavirus pandemic has placed extraordinary weight on the global burden of stress [72,73] with healthcare workers also facing tremendous psychosocial and physiologic stress burden [74,75]. Night shift workers (predominately nurses) who present with significant disruption in diurnal cortisol levels are therefore more susceptible to immune compromise [76]. Accessible GSIs to curb stress-related health risks during this unusually stressful time should be thoroughly studied to best meet a growing demand.

All but three studies included in this review assessed mental health outcomes alongside physiologic indicators of stress [37,52,53]. This suggests that the causal connection or at a minimum, the associations between psychological state and physiologic outcomes should be studied further. There is a great opportunity to study the impact of perceived stress on the more nuanced and complex analysis of cortisol determines how affinity for nature, or familiarity for natural surroundings, impact cortisol levels. Along with gathering more data on cortisol levels over time, procedures for controlling potential confounding variables should be built into future experimental research designs. Results of these studies in this scoping review demonstrate that GSIs may have the potential for a profound impact on reducing stress as measured by cortisol, yet the generalizability of the results is complicated by the vast and varied nature of study protocol and data interpretation. This scoping review demonstrates that deeply codified GSIs based on well-known theoretical frameworks may mitigate cortisol production and thereby reduce physiologic stress burden for persons across the lifespan.

## Figures and Tables

**Table 1:** Databases and keyword search terminology.

Databases	Keyword Search Terms
CINAHL* Plus with Full-Text PubMed Scopus	(“greenspace” OR “green space” OR “green care” OR “greencare” OR “nature therap*” OR “wilderness therap*” OR “outdoors behavi*ral healthcare” OR “outdoors behavi*ral therap*” OR “forest bathing” OR “shinrin yoku” OR “shinrinyoku” OR “horticultur* therap*” OR “therapeutic horticulture” OR “green exercise” OR “ecotherap*” OR “conservation therap*” OR “care farm*” AND “cortisol”) AND intervention

* Cumulative Index of Nursing and Allied Health Literature.

**Table 2:** Literature Matrix.

Article & Location	Sample	Design	Intervention	Cortisol Measures	Results
**Horticulture Therapy (HT)**					
Detweiler, Self [36] U.S.A	*n* = 24, *n* = 20 (HT), *n* = 18 (OT) Mean age 46.4 yrs. (SD 11.9) 0.03% female	Quasi-experimental, single arm	Setting(s): outdoor gardens (intervention), residential facility (control). Duration: 15, 60 min sessions over 3 weeks.	Salivary cortisol. Collected by research team. Time standardization. Measured at weeks 1, 2, and 3.	No between groups comparison due to sampling inaccuracy. Nonsignificant downward cortisol trend over time.
Han, Park [37] South Korea	*n* = 28, *n* =14 Mean age 80.1 yrs. (SD 2.9) 8.6% female	Quasi-experimental, single arm	Setting(s): outdoor farm garden space (intervention), control setting unclear. Duration:10, 90 min sessions over 2 months.	Salivary cortisol Collected by research team Time standardized Measured at pre-test and post-test.	Significant cortisol decrease over time (*M* 7.56 - *M* 3.80 (*p* < 0.05). No significant cortisol change in control group. No significant between group difference.
Ng, Sia [38] Singapore	*n* = 59, *n* =29 (treatment), *n* = 30 (waitlist control) Mean age 67.1 yrs. (SD 4.31) 78% female	Randomized wait-list controlled trial	Setting(s): outdoor parks, gardens, and nature reserves (intervention), waitlist (control) Duration: 15 hr. sessions for 3 months, repeated waitlist (6 months).	Plasma cortisol (fasting, venous blood). Collected by research team.Time standardized Measured at baseline, 3 months, and 6 months.	No significant effects of time, group, or time × group interactions on plasma cortisol.
Van Den Berg and Custers [39] The Netherlands	*n* = 30, *n* = 14 (gardening condition), *n* = 16 (reading condition) Mean age 57.6 (38 – 79) yrs. 73.3% female	Quasi-experimental, single arm	Setting(s): personal outdoor and indoor gardens (intervention), reading (control) Duration: 30 min, “stressful task” for 25 additional minutes after baseline- before experimental activity.	Salivary cortisol. Collected by research team. Time not standardized. Measured at baseline, pre-stressful task, post- stressful task, during condition, postcondition over 2-weeks.	Stressful task non-significantly increased cortisol across both conditions compared to baseline. Significant decrease in cortisol post-stressor to post-condition in intervention (*p* < 0.001), and control group (*p* < 0.05). Significant condition to post-condition cortisol decrease in intervention group, (*p* < 0.05), but not control group.
**Forest Bathing (FB) (Shinrin yoku) Active or Passive**					
Kobayashi, Song [40] Japan	*n* = 74 Mean age 22.4 yrs. (SD. 1.8) 0% female	Quasi-experimental crossover	Active (walking) Settings(s): 7 forests and urban centers, ratio unclear. Duration: 25 min in an urban and forested site per participant (two site visits) over two days.	Salivary cortisol. Collector undisclosed. No time standardization Measured pre-test and post-test.	Significant interaction effect between setting X walking (*p* < 0.001). Significantly lower cortisol after walking in FB group than urban (*p* < 0.001). Significant pre-post decrease in cortisol (*p* < 0.001), but not urban walking.
Lee, Park [41] Japan	*n* = 12 Mean age 21.2 yrs. (SD – 0.9) 0% female	Quasi experimental crossover	Passive (sitting) Setting(s): Forested region, urban street view Duration: 15 min at each site over two days.	Salivary cortisol. Collector undisclosed. Time standardization. Measured baseline, pre- and post.	Baseline cortisol significantly lower in FB group than urban (*p* < 0.05). Just before stimuli period, cortisol levels lower in the FB group than urban (*p* < 0.01). No significant differences in cortisol levels before and after stimuli period.
Mao, Lan [42] China	*n* = 20, *n* = 10 Mean age 20.79 yrs. (SD = 0.54) 0% female	Randomized controlled trial	Active (walking). Setting(s): Forest site (intervention), city site (control). Duration: 1.5 h with 10 min rest twice a day for two days.	Serum cortisol. Collected by research team. Time standardization. Measured at baseline, pre-test and post-test.	Significantly lower cortisol in FB group than city-site post-intervention (*p* < 0.05). Exact levels for cortisol not reported.
Sung, Woo [43] South Korea	*n* = 56, *n* = 28 Mean age 66 yrs. (SD 7) 0% female	Quasi experimental, single arm	Forest therapy program (FTP). Setting(s): two recreational forests. Duration: 3 days over 8 weeks.	Salivary cortisol. Researchers collected samples. Time standardization not reported. Measured at baseline and 8 weeks postexposure.	Significant pre to posttest difference, cortisol reduction larger in FB compared to the control group 0.03 (−0.02 to 0.08) vs. −0.03 (−0.11 to 0.01) μg/dL, (*p* < 0.05).
**Greenspace Exposure (GE) Active or Passive**					
Beil and Hanes [44] U.S.A.	*n* = 15 Mean age 42.3 (20 – 61) yrs. 46.7% female 100% Non- Hispanic White (NHW)	Quasi experimental, multi-arm, cross-over	Passive (sitting). Setting(s): Very Natural, Mostly Natural, Mostly Built, Very Built. Duration: 20 min. per setting, 120 min total over one month.	Salivary cortisol. Participants self-collected salivary. samples with oral swabs. Time standardized per setting. Measured pre-test and post-test.	Non-significant trend of decrease in cortisol post exposure in Very Natural and Mostly Natural settings, larger decreased in cortisol for the Mostly Built setting compared to the Very Built setting. No gender differences detected.
Grazuleviciene, Vencloviene [45] Lithuania	*n* = 20, *n* = 10 Mean age 62.3 yrs. (SD – 12.6) 35% female	Randomized controlled trial	Active (outdoor walking). Setting(s): green, forested park (intervention), urban street (control). Duration: 30 min once a day for 7 days.	Salivary cortisol. Affect (PANAS). Collector undisclosed. Time standardized. Measured 3 times per day: baseline, immediately following the exposure (1 min after walking in either environment), 60 min after the exposure.	Significant correlation between negative affect (NA) and higher mean cortisol levels across all participants (*p* < 0.05). Cortisol levels not significantly correlated with positive affect (PA). SBP and cortisol negatively associated (*p* < 0.1). DBP and heart rate and cortisol slope over 7 days (*p* < 0.05). No significant between group differences.
Mokhtar, Abdul Aziz [46] Malaysia	*n* = 20, Mean age 23.1 yrs. 0% female	Quasi-experimental, single arm crossover	Active (walking) Settings: Urban greenspace (UGS) (intervention), city center (control). Duration: 20 min, twice in one day.	Salivary cortisol. Collector undisclosed. No time standardization. Measured pre-test and post-test.	Significantly greater decrease in cortisol pre to post-test in UGS than city group, (UGS: 0.89 ± 0.55, city: 2.33 ± 1.04, *p* < 0.05). Significantly increased cortisol in city group from pre: 1.75 ± 1.00 μg/dl, to post 2.33 ± 1.04 μg/dl (*p* < 0.05).
Olafsdottir, Cloke [47] Iceland	*n* = 67, *n* = 20 (nature setting), 30 (gym), 30 (TV) Mean age 24.4 yrs. (SD 2.61) 8.7% female	Mixed-method factorial-multi-arm design.	Active (walking, exercising). Setting(s): Spruce forest (nature group), indoor gym (treadmill group), indoor laboratory (video group). Duration: 40 min in each condition over 76 min of data collection over 2 months.	Salivary cortisol. Researcher collected. No time standardization.Measured pre- post intervention and after *The Socially* *EvaluatedCold-Pressor Test* (SECPT) was introduced.	Cortisol significantly decreased from pre to post all groups (*p* < 0.001), returned to baseline after SECPT (*p* < 0.001). Significantly lower cortisol in nature group compared to the video group post-intervention (*p* = 0.046). Exact cortisol values not reported.No gender differences reported.
Razani, Morshed [48] U.S.A.	*n*= 154 (78 dyads), *n* = 50 (supported group), Parent mean age, 38.95 yrs. Child mean age 8.8 yrs. Parent 87% female Child 49% female 5% NHW	Quasi- experimental	Active (play), Setting(s): regional green park (supported park prescription group), no setting disclosed for independent park prescription group. Duration: 1–3 park outings once a month for 3 months.	Salivary cortisol. Study staff collected samples. No time standardization. Measured baseline, 1 month and 3 months post intervention.	Cortisol decreased significantly from baseline M = 0.18, SD = 0.13, to 3 months, M = 0.12, SD = 0.07, (*p* = 0.0241), CI = 0.05, 0.65. All other analyses were non-significant.
Tyrväinen, Ojala [49] Finland	*n* = 77, Mean age 47.64 yrs. (SD 8.68) 92.2% Female.	Quasi-experimental, multi-arm crossover	Active (walking) and passive (sitting). Setting(s): urban park, large urban woodland (interventions), city center (control). Duration: roughly 3-h per month over 10 months.	Salivary cortisol. Collector undisclosed. No time standardization. Measured pre, during, and post-intervention.	Significant decrease in cortisol between pre and post exposure (*p* < 0.01) in all groups. No significant between groups differences.
Calogiuri, Evensen [50] Norway	*n* = 14, *n* = 6 (green exercise), *n* = 7 (gym). Mean age 49 years (SD – 8) Female 50%	Quasi-experimental single arm crossover	Setting(s): Indoor gym (indoor group, control) and an outdoor forested park (outdoor group, intervention). Duration: 3 days over two-weeks with longitudinal follow-up at week 10.	Serum cortisol. Time standardized: participants self-collected saliva forCAR upon awakening, 15 min, 30 min.Nurses collected serum samples for CAR post exposure in the AM.Measured pre, post, 3 time points: baseline, afternoon and evening for 3 days.	Significantly lower postexposure cortisol for intervention group compared to control (*p* = 0.04). No significant differences between groups found for CAR AUC_G_ and serum cortisol. Non-significant higher AM cortisol levels and more rapid decrease 30 min. post AM than control.
**Outdoor Activity Programming (OAP)**					
Chang, Davidson [51] U.S.A.	*n* = 33, *n* = 3 (kayaking), *n* = 3 (backpacking), *n* = 4 (canoeing) Mean age 20.67 yrs. (SD 1.45) 66.7% female	Quasi experimental non-equivalent groups mixedmodel	Settings(s): field trip courses in three independent groups: canoeing (*n* = 9), backpacking (*n* = 16) kayaking (*n* = 8). Duration: 3 days over 2 weeks.	Salivary cortisol. Collected by research team. Not time standardized. Measured baseline, pretest and post-test.	Nonsignificant trend of decreasing cortisol over time in all groups. Significant decrease in cortisol from pre to posttest in all groups (*p* < 0.05) No significant interaction or main effect of sex and time, or activity and time on cortisol.
Dettweiler, Becker [52] Germany	*n* = 48 Mean age 11.6 yrs. 38% female (forest) 36% female(control)	Quasi-experimental Prospective longitudinal single-arm	Setting(s): forested park (outdoor learning intervention) and indoor classroom (control) Duration: 1 day each week for 9 months.	Salivary cortisol. Collector undisclosed. Time standardized. Measured at 8:30, 10:30 AM, and 12:30 PM, intervention day, 1 day per week, during school year	Intervention group significant greater overall decline of cortisol than controls, (*p* < 0.01). Nonsignificant trend of intervention group lower cortisol levels in spring compared to other seasons (*p* = 0.05).
Niedermeier, Grafetstätter [53] Austria	*n* = 42, Mean age: 32 yrs. (SD 12). 48% Female.	Quasi experimental multi-arm crossover	Setting(s): Mountain region in a forest, indoor treadmill walking in a gym (interventions), computer room at a local University (control). Duration: 3 h in each condition over 14 days.	Salivary cortisol. Collector undisclosed. No time standardization. Measured pre-test and post-test.	Cortisol decreased significantly in all conditions over time (*p* < 0.001). Cortisol decreased more pre to post test in both intervention groups compared to control (*p* < 0.05). No significant difference between intervention groups.
